# Transmission of allergen-specific IgG and IgE from maternal blood into breast milk visualized with microarray technology

**DOI:** 10.1016/j.jaci.2014.08.041

**Published:** 2014-11

**Authors:** Heidrun Hochwallner, Johan Alm, Christian Lupinek, Catharina Johansson, Axel Mie, Annika Scheynius, Rudolf Valenta

**Affiliations:** a Division of Immunopathology, Department of Pathophysiology and Allergy Research, Medical University of Vienna, Austria; b Department of Clinical Science and Education, Södersjukhuset, Karolinska Institutet, Stockholm, Sweden; c Sachs' Children and Youth Hospital, Södersjukhuset, Stockholm, Sweden; d Department of Medicine Solna, Translational Immunology Unit, Karolinska Institutet and University Hospital, Stockholm, Sweden

To the Editor:

Data from experimental animal models have previously shown that allergen-specific IgG antibodies are transmitted from the mother to the offspring via breast milk^1^, ^2^ and have provided evidence that the transmitted allergen-specific IgG antibodies protect specifically against allergic sensitization.^2^ In some studies, it has been demonstrated for humans that breast-feeding has a prophylactic effect against atopic disease but there are also reports arguing against this and the underlying mechanisms are not known.^3^ There is evidence that IgG antibodies against bacterial antigens (ie, pneumococcal antigens) are transferred from the blood of mothers into their breast milk.^4^ Furthermore, it was possible to detect IgA and IgG against different food antigens in human serum, saliva, colostrums, and milk samples.^5^ Another study found that total IgE levels in breast milk and blood were associated but allergen-specific IgE was not analyzed.^6^

With the FP7-funded European Union research program Mechanisms of the Development of ALLergy (MeDALL; http://medall-fp7.eu/), we have recently developed a microarray containing a large number of purified natural and recombinant respiratory, food, and insect allergens that allows highly sensitive measurement of allergen-specific IgE and IgG levels with minute amounts of blood.^7^ A major advantage of the microarray technology is that it allows one to measure antibody reactivities toward a large panel of different allergens. Here, we investigated whether the MeDALL chip is suitable for (1) the measurement of allergen-specific IgG and IgE levels in human breast milk samples, (2) whether there is a transmission of allergen-specific antibodies from blood into breast milk, and (3) whether the reactivity profile of allergens recognized by antibodies in blood and milk is similar. For this purpose, we analyzed plasma and breast milk samples from sensitized (n = 23) and nonallergic mothers (n = 6) from the ALADDIN birth cohort.^8^ None of the mothers was on allergen-specific immunotherapy. Maternal blood samples were collected in the period around delivery (−1 to +2 months), and the breast milk samples were obtained 2 months after delivery. The study was approved by the local Research Ethical Committee, and written informed consent was obtained from all families.

The breast milk samples were centrifuged for 10 minutes at 2500*g* before use to remove the lipids. For comparison of IgG titers in plasma and breast milk, the plasma samples were diluted 1:50, 1:100, 1:200, and 1:400 before analysis. Microarrays were incubated with 30 μL of the plasma dilutions or undiluted breast milk samples and allergen-specific IgG and IgE antibodies were detected with fluorophore-conjugated anti-IgG and anti-IgE antibodies, respectively.^7^ The fluorescence intensities were measured with a biochip scanner. Results were expressed in ISAC standardized units (Thermofisher, Uppsala, Sweden). Correlation coefficients were calculated with SPSS.

Detailed analysis of allergen-specific IgG and IgE levels in plasma and breast milk samples indicated that allergen-specific IgG antibodies are transmitted from the blood into breast milk in a highly specific manner and that breast milk IgG mirrored the profile of IgG reactivity in the blood (see Fig E1 in this article's Online Repository at www.jacionline.org). A comparison of allergen-specific IgG levels measured in 4 plasma dilutions with that of undiluted breast milk samples (Fig 1) indicated that allergen-specific IgG levels in breast milk were approximately 200- to 400-fold lower than in plasma. Allergen-specific IgG reactivities in plasma and breast milk were significantly correlated; for the 1:200 dilution, Spearman correlation coefficient was 0.608 (*P* < .001) and for the 1:400 dilution, Spearman correlation coefficient was 0.604 (*P* < .001) (Fig 2). Detailed results are displayed for each allergen in the heat map (Fig E1). For the vast majority of allergens, plasma- and milk-derived IgG antibody reactivities were correlated. However, in certain instances (eg, milk allergens recognized by donor 3), specific IgGs were high in plasma but did not appear in milk; in some other cases, allergen-specific IgG was detected only in milk but not in plasma (Fig E1). Possible explanations for lack of allergen-specific IgG binding in milk are that certain antigens are present in milk and inhibit IgG binding and/or low affinity/avidity of IgG may prevent binding despite high titers in blood. In fact, the presence of certain respiratory and food allergens in breast milk has been recently demonstrated.^9^, ^10^ However, milk-specific IgG may appear because of local IgG production without corresponding IgG in blood.

**Fig 1:**
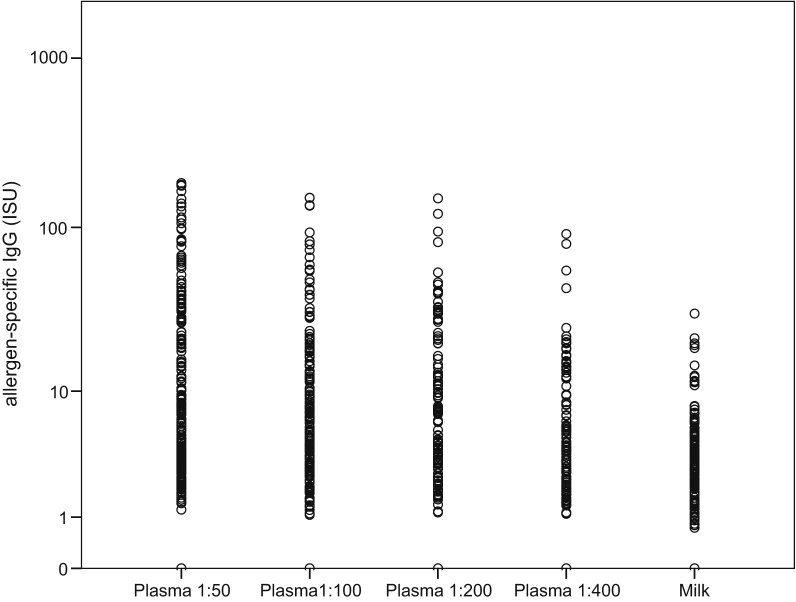
Comparison of allergen-specific IgG levels (ISAC standardized unit [ISU]) measured in different plasma dilutions of 4 mothers with allergen-specific IgG levels in their breast milk samples.

**Fig 2:**
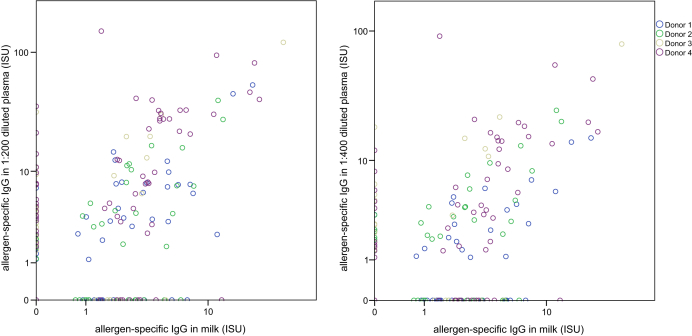
Correlation of allergen-specific IgG levels (ISAC standardized unit [ISU]) in plasma samples (*left*: dilution 1:200; *right*: dilution 1:400) from 4 mothers (*the donors are labeled in different colors*) with allergen-specific IgG levels in their corresponding undiluted breast milk samples.

The presence of allergen-specific IgG in breast milk may be due to specific transmission of allergen-specific IgG from the blood of mothers into their breast milk or eventually because of local production. For transmission, active transport by the IgG receptor FcRn, which in fact is expressed in the human mammary gland,^11^ and/or transudation from blood into the mammary glands, as was found for allergen-specific IgG in mucosal fluids, may be considered.^12^ Interestingly, for mothers with high levels of allergen-specific IgE in their plasma (donors 1, 9, 13, 15, 16, and 19), we also found that specific IgE antibodies were present in breast milk. In all but 1 case (donor 3, Phl p 5–specific IgE), the presence of allergen-specific IgE was associated with levels of IgE in the blood. The allergen-specific IgE reactivity profiles in blood and breast milk are summarized in detail in Figs E1 and E2 in this article's Online Repository at www.jacionline.org.

In none of the 6 nonallergic mothers (donors 24-29), who served as negative controls, allergen-specific IgE was detected in plasma or in breast milk, demonstrating the specificity of IgE test results (Fig E2).

Our results thus demonstrate that allergen-specific IgG and IgE antibodies with similar specificity are present in blood and in breast milk. Furthermore, our study shows that the MeDALL allergen chip is suitable for the measurement of allergen-specific IgG and IgE antibodies not only in blood but also in breast milk, which opens the possibility to study the transmission of allergen-specific IgG antibodies from mothers to offsprings on the development of allergic sensitization in birth cohorts.

## Supplementary data

Legends for Figures E1 and E2

Supplementary Figure E1

Supplementary Figure E2
