# Supplementary material for: Transmission of allergen-specific IgG and IgE from maternal blood into breast milk visualized with microarray technology
Source: J Allergy Clin Immunol. 2014 Nov;134(5):1213–5. doi: 10.1016/j.jaci.2014.08.041 (PMC4220005; doi:10.1016/j.jaci.2014.08.041)
Supplement: Legends for Figures E1 and E2 [file mmc1.docx]

FIG E1. Heat map of allergen-specific IgG and IgE reactivities measured in different plasma dilutions for 4 mothers (donors 1-4) and in their corresponding undiluted breast milk samples. A color code for the antibody levels (ISU) is shown. Cutoff for IgG and IgE: 0.1 ISU. *ISU*, ISAC standardized unit; *M*, milk; *Pl*, plasma.

FIG E2. Heat map of allergen-specific IgE reactivities measured in undiluted plasma samples of 19 sensitized (donors 5-23) and 6 nonallergic mothers (donors 24-29) and in the corresponding undiluted breast milk samples. A color code for the antibody levels (ISU) is shown. Cutoff for IgG and IgE: 0.1 ISU. *ISU*, ISAC standardized unit; *M*, milk; *Pl*, plasma.
