# Supplementary material for: Transmission of allergen-specific IgG and IgE from maternal blood into breast milk visualized with microarray technology
Source: J Allergy Clin Immunol. 2014 Nov;134(5):1213–5. doi: 10.1016/j.jaci.2014.08.041 (PMC4220005; doi:10.1016/j.jaci.2014.08.041)
Supplement: Supplementary Figure E1 [file mmc2.pdf]

Supplementary Figure E1

Donor 1	PI 1:50	PI 1:100	PI 1:200	PI 1:400	M	PI	PI 1:50	PI 1:100	PI 1:200	PI 1:400	M
Method	IgG					IgE					
Act d 2	0	0	0	0	0	0	0	0	0	0	0
Act d 5	0	0	0	0	0	0	0	0	0	0	0
Act d 8	0	0	0	0	0	0	0	0	0	0	0
Aln g 1	0	0	0	0	0	0	0	0	0	0	0
Alt a 1	0	0	0	0	0	0	0	0	0	0	0
Alt a 6	0	0	0	0	0	0	0	0	0	0	0
Amb a 1	0	0	0	0	0	0	0	0	0	0	0
V Ana o 1	0	0	0	0	0	0	0	0	0	0	0
Ana o 2	0	0	0	0	0	0	0	0	0	0	0
V Ana o 2	0	0	0	0	0	0	0	0	0	0	0
V nAna o 2	0	0	0	0	0	0	0	0	0	0	0
V Ana o 3	0	0	0	0	1.48	0	0	0	0	0	0
Ani s 3	0	0	0	0	1.25	0	0	0	0	0	0
Api m 1	0	0	0	0	0	0.45	0.56	0.55	0.42	0.33	0
Api m 4	0	0	0	0	0	0	0	0	0	0	0
Ara h 1	0	0	0	0	0	0	0.96	4.33	0.56	0	0
Ara h 2	0	0	0	0	0	0	0	0	0	0	0
Ara h 3	0	0	0	0	0	0	0	0	0	0	0
Ara h 6	0	0	0	0	0	0	0	0	0	0	0
Ara h 8	0	0	0	0	0	0	0	0	0	0	0
Ara h 9	0	0	0	0	0	0	0	0	0	0	0
Art v 1	5.05	2.87	1.13	0	1.08	22.16	1.71	0.88	0.56	0.30	0
Art v 3	0	0	0	0	0	0	0	0	0	0	0
Asp f 1	0	0	0	0	0	0	0	0	0	0	0
Asp f 3	0	0	0	0	0	0	0	0	0	0	0
Asp f 6	0	0	0	0	0	0	0	0	0	0	0
Ber e 1	0	0	0	0	0	0	0	0	0	0	0
Bet v 1	23.58	9.25	7.23	1.12	5.25	101.93	10.68	4.74	3.54	1.78	0.69
Bet v 2	31.41	15.49	7.61	2.72	7.57	8.89	1.13	0.81	0.36	0	0
Bet v 4	0	0	0	0	0	0.52	0	0	0	0	0
Bla g 1	0	0	0	0	0	0	0	0	0	0	0
Bla g 2	8.99	6.38	3.35	1.93	1.81	0	0	0	0	0	0
Bla g 5	20.09	0	0	0	0	0.23	0	0	0	0	0
Bla g 7	0	0	0	0	3.14	0	0	0	0	0	0
Blo t 5	0	0	0	0	0	0	0	0	0	0	0
Bos d 4	0	0	0	0	0	0	0	0	0	0	0
V Bos d 4	0	0	0	0	0	0	0	0	0	0	0
Bos d 5	3.71	0	0	0	0.92	0	0	0	0	0	0
V Bos d 5	37.66	21.94	9.83	3.71	5.35	0	0	0	0	0	0
Bos d Lactoferrin	32.92	14.67	7.56	4.19	6.25	0	0	0	0	0	0
Bos d 8	0	0	0	0	0	0	0	0	0	0	0
V Bos d 8	8.33	4.22	2.05	0	1.52	0	0	0	0	0	0
aS1-casein	0	0	0	0	0	0	0	0	0	0	0
aS2-casein	0	0	0	0	0	0	0	0	0	0	0
b-casein	0	0	0	0	0	0	0	0	0	0	0
K-casein	4.83	3.08	0	0	0	0	0	0	0	0	0
Transferrin	36.68	22.89	12.26	0	5.23	0	0	0	0	0	0
Bos d 6	15.40	5.53	3.31	0	5.31	0	0	0	0	0	0
V BSA	0	0	0	0	3.49	0	0	0	0	0	0
Can f 1	0	0	0	0	0	0	0	0	0	0	0
Can f 2	0	0	0	0	0	0	0	0	0	0	0
Can f 3	0	0	0	0	0	0	0	0	0	0	0
V Can f 4	0	0	0	0	0	0	0	0	0	0	0
Can f 5	0	0	0	0	0	1.17	0	0	0	0	0
V Can f 5	0	0	0	0	0	0	0	0	0	0	0
V Can f 6	0	0	0	0	0	0	0	0	0	0	0
Che a 1	0	0	0	0	0	0	0	0	0	0	0
Cla h 8	0	0	0	0	1.42	0	0	0	0	0	0
Cor a 1.0401	1.77	1.06	0	0	1.52	26.73	1.34	0.69	0.46	0.27	0
Cor a 8	0	0	0	0	5.64	0	0	0	0	0	0
Cor a 9	0	0	0	0	0	0	0	0	0	0	0
Cry j 1	13.58	6.54	3.61	1.38	2.39	6.81	0.57	0	0	0	0
Cyn d 1	48.22	29.21	12.55	4.88	2.01	145.57	10.47	5.91	3.36	1.78	0.66
Cup a 1	21.44	10.37	4.51	1.74	2.14	9.48	0.88	0.63	0	0	0
Der f 1	2.33	0	0	0	0	0	0	0	0	0	0
Der f 2	0	0	0	0	0	0	0	0	0	0	0
Der p 1	0	0	0	0	0	0	0	0	0	0	0
Der p 2	0	0	0	0	0	0	0	0	0	0	0
V Der p 4	0	0	0	0	0	0	0	0	0	0	0
V Der p 5	1.90	0	0	0	0	0	0	0	0	0	0
V Der p 7	0	0	0	0	1.08	0	0	0	0	0	0
Der p 10	0	0	0	0	0	0	0	0	0	0	0
V Der p 11	0	0	0	0	0	0	0	0	0	0	0
V Der p 14	0	0	0	0	0	0	0	0	0	0	0
V Der p 15	3.83	0	0	0	0	0	0	0	0	0	0
V Der p 18	0	0	0	0	0	0	0	0	0	0	0
V Der p 21	0	0	0	0	0	0	0	0	0	0	0
V Der p 23	0	0	0	0	0	0	0	0	0	0	0
V clone 16	0	0	0	0	0	0	0	0	0	0	0

Donor 1	PI 1:50	PI 1:100	PI 1:200	PI 1:400	M	PI	PI 1:50	PI 1:100	PI 1:200	PI 1:400	M
Method			IgG					IgE			
Equ c 1	0	0	0	0	0	0.46	0	0	0	0	0
Equ c 3	0	0	0	0	0	0.84	0	0	0	0	0
Fag e 2	0	0	0	0	0	0	0	0	0	0	0
Fel d 1	4.43	2.50	0	0	0	2.94	0.79	0	0	0	0
Fel d 2	0	0	0	0	0	4.14	0	0	0	0	0
Fel d 4	2.88	1.89	1.14	0	0	0	0	0	0	0	0
Gad c 1	0	0	0	0	0	0	0	0	0	0	0
Gal d 1	15.90	7.78	3.70	1.43	1.01	0	0	0	0	0	0
Gal d 2	2.22	1.46	0	0	0	0	0	0	0	0	0
Gal d 3	0	0	0	0	0	0	0	0	0	0	0
Gal d 5	0	0	0	0	1.94	0	0	0	0	0	0
Gly m 4	0	0	0	0	0	0	0	0	0	0	0
Gly m 5	0	0	0	0	0	0	0	0	0	0	0
Gly m 6	0	0	0	0	0	0.32	0	0	0	0	0
Hev b 1	0	0	0	0	0	0	0	0	0	0	0
Hev b 3	0	0	0	0	0	0	0	0	0	0	0
Hev b 5	0	0	0	0	1.38	0	0	0	0	0	0
Hev b 6.01	0	0	0	0	0	0	0	0	0	0	0
Hev b 8	147.53	93.29	53.88	15.16	19.50	24.82	3.35	2.01	0.98	0.54	0.42
Jug r 1	0	0	0	0	0	0.76	0	0	0	0	0
Jug r 2	33.46	21.33	7.98	0	2.30	1.65	0.67	0.47	0	0	0
Jug r 3	0	0	0	0	0	0	0	0	0	0	0
Lep d 2	0	0	0	0	0	0	0	0	0	0	0
Mal d 1	20.74	11.98	6.40	0	3.45	30.6	1.84	1.03	0.61	0.39	0.37
Mer a 1	33.21	23.90	6.29	6.87	7.93	6.32	0.92	0.87	0.46	0.21	0
Mus m 1	0	0	0	0	0	0	0	0	0	0	0
MUXF3	3.36	0	0	0	0	0	0	0	0	0	0
Ole e 1	2.04	0	0	0	0	0.50	0	0	0	0	0
Ole e 7	0	0	0	0	0	0	0	0	0	0	0
Ole e 9	20.85	12.15	3.50	2.33	4.05	0	0	0	0	0	0
Par j 2	3.17	0	0	0	7.23	0	0	0	0	0	0
Pen m 1	0	0	0	0	0	0	0	0	0	0	0
Pen m 2	0	0	0	0	0	0	0	0	0	0	0
Pen m 4	1.41	0	0	0	0	14.19	0	0	0	0	0
Phl p 1	10.44	5.09	2.94	1.09	2.81	138.7	13.69	7.60	4.55	2.51	0.72
Phl p 2	0	0	0	0	0	1.56	0	0	0	0	0
Phl p 4	44.07	28.67	14.76	4.30	1.94	3.43	0.72	0.59	0	0	0
Phl p 5	10.12	4.94	1.60	0	0	106.67	4.13	3.31	1.83	0.88	0.47
Phl p 6	3.24	1.96	0	0	0	40.29	2.96	1.78	0.91	0.48	0
Phl p 7	0	0	0	0	0	0.66	0	0	0	0	0
Phl p 11	0	0	0	0	0	1.24	0.44	0	0	0	0
Phl p 12	1.21	1.31	0	0	0	0.69	0	0	0	0	0
V Pis v 3	0	0	0	0	0	0	0	0	0	0	0
Pla a 1	0	0	0	0	0	0.50	0	0	0	0	0
Pla a 2	27.28	14.76	7.71	2.68	2.05	4.95	0.41	0.43	0	0	0
Pla a 3	0	0	0	0	0	0	0	0	0	0	0
Pla l 1	0	0	0	0	0	0	0	0	0	0	0
Pol d 5	0	0	0	0	0	29.35	1.26	0.79	0.35	0	0
Pru p 1	0	0	0	0	0	17.08	0	0	0	0	0
Pru p 3	11.56	10.03	2.39	5.46	11.49	0	0	0	0	0	0
V Pru du 3	0	0	0	0	0	0	0	0	0	0	0
V Pru du 4	114.64	73.39	45.54	14.05	14.59	15.20	3.06	1.87	0.93	0.59	0.31
V Pru du 6	1.81	1.20	0	0	3.52	0	0	0	0	0	0
V Pru du 6.01	0	0	0	0	0	0	0	0	0	0	0
V Pru du 6.02	0	0	0	0	0	0	0	0	0	0	0
Sal k 1	3.54	1.92	0	0	0	0.39	0	0	0	0	0
Ses i 1	0	0	0	0	0	0	0	0	0	0	0
Tri a 14	0	0	0	0	0	0	0	0	0	0	0
Tri a 19.0101	0	0	0	0	0	0	0	0	0	0	0
Tri a aA_TI	19.00	13.29	7.07	3.34	0	0	0	0	0	0	0
V Tri a 36 191	0	0	0	0	0	0	0	0	0	0	0
V Tri a 36	0	0	0	0	0	0	0	0	0	0	0
V m43	7.82	4.05	2.43	1.13	0.79	0.92	0	0	0	0	0
V m82	0	0	0	0	0	0	0	0	0	0	0
Serine	5.39	2.73	1.37	0	0	0	0	0	0	0	0
Thioredoxin	0	0	0	0	0	0	0	0	0	0	0
Glutathione	0	0	0	0	0	0	0	0	0	0	0
peroxiredoxin	0	0	0	0	0	0.53	0	0	0	0	0
Profilin	39.03	16.69	7.87	5.81	3.67	8.05	0.98	0.66	0	0	0
Dehydrin	3.41	0	0	0	0	1.42	0	0	0	0	0
purothionin	1.96	0	0	0	0	0	0	0	0	0	0
LTP	0	0	0	0	0	0	0	0	0	0	0
V Ves v 1	0	0	0	0	0	0	0	0	0	0	0
Ves v 5	0	0	0	0	0	0	0	0	0	0	0
V Ves v 5	0	0	0	0	0	0	0	0	0	0	0

IgE

0.1-0.99	
1-14.99	
≥ 15	

IgG

0.1-0.99	
1-14.99	
≥ 15	

Donor 2	PI 1:50	PI 1:100	PI 1:200	PI 1:400	M	PI	M
Method	IgG					IgE	
Act d 2	0	0	0	0	0	0	0
Act d 5	0	0	0	0	0	0	0
Act d 8	0	0	0	0	0	0	0
Aln g 1	0	0	0	0	0	0	0
Alt a 1	0	0	0	0	0	0	0
Alt a 6	0	0	0	0	0	0	0
Amb a 1	0	0	0	0	0	0	0
V Ana o 1	0	0	0	0	0	0	0
Ana o 2	0	0	0	0	0	0	0
V Ana o 2	0	0	0	0	0	0	0
V nAna o 2	11.45	5.43	2.91	1.86	1.23	0	0
V Ana o 3	4.15	0	0	0	2.12	0	0
Ani s 3	4.65	0	0	0	3.50	0	0
Api m 1	0	0	0	0	1.02	0.21	0
Api m 4	0	0	0	0	0	0	0
Ara h 1	0	0	0	2.05	1.09	0	0
Ara h 2	0	0	0	0	0	0	0
Ara h 3	0	0	0	0	0	0	0
Ara h 6	0	0	0	0	0	0	0
Ara h 8	0	0	0	0	0	0	0
Ara h 9	14.31	3.83	1.82	1.48	0	0	0
Art v 1	4.12	0	0	0	0	0	0
Art v 3	0	0	0	0	0	0	0
Asp f 1	0	0	0	0	0	0	0
Asp f 3	0	0	0	0	0	0	0
Asp f 6	0	0	0	0	0	0	0
Ber e 1	0	0	0	0	0	0	0
Bet v 1	0	0	0	0	0	0	0
Bet v 2	0	0	0	0	0	0	0
Bet v 4	0	0	0	0	0	0	0
Bla g 1	9.05	7.70	5.06	3.97	1.13	0	0
Bla g 2	66.25	42.31	27.87	20.41	12.56	0	0
Bla g 5	63.02	5.25	0	0	1.81	0	0
Bla g 7	5.87	0	0	0	0.73	0	0
Blo t 5	4.03	3.01	0	0	0	0	0
Bos d 4	0	0	0	0	0	0	0
V Bos d 4	0	0	0	0	0	0	0
Bos d 5	36.53	17.65	11.23	3.97	2.55	0	0
V Bos d 5	133.17	82.85	40.17	24.93	11.65	0.15	0
Bos d Lactoferrin	8.41	3.03	2.10	0	0	0	0
Bos d 8	0	0	0	0	0	0	0
V Bos d 8	17.86	10.45	4.28	3.19	2.01	0	0
aS1-casein	0	1.77	0	0	0	0	0
aS2-casein	3.45	3.90	1.83	0	2.36	0	0
b-casein	0	0	0	0	0	0	0
K-casein	7.49	4.30	1.65	0	0	0	0
Transferrin	10.20	5.76	2.29	0	0	0	0
Bos d 6	7.01	3.21	0	0	0.97	0	0
V BSA	4.29	0	0	0	0	0	0
Can f 1	0	0	0	0	0	0	0
Can f 2	0	0	0	0	0	0	0
Can f 3	2.92	0	0	0	0	0	0
V Can f 4	0	0	0	0	0	0	0
Can f 5	3.09	0	0	0	0	0	0
V Can f 5	6.48	0	0	0	0	0	0
V Can f 6	0	0	0	0	0	0	0
Che a 1	0	0	0	0	0	0	0
Cla h 8	7.39	0	0	0	0	0	0
Cor a 1.0401	0	0	0	0	0	0	0
Cor a 8	46.38	16.85	7.35	8.19	8.01	0	0
Cor a 9	5.07	1.59	0	0	0	0	0
Cry j 1	0	0	0	0	0	0	0
Cyn d 1	8.21	0	0	0	0	0.53	0
Cup a 1	5.46	0	0	0	0	0	0
Der f 1	0	0	0	0	0	0	0
Der f 2	0	0	0	0	0	0	0
Der p 1	0	0	0	0	0	0	0
Der p 2	0	0	0	0	0	0	0
V Der p 4	0	0	0	0	0	0	0
V Der p 5	0	0	0	0	0	0	0
V Der p 7	0	0	0	0	0	0	0
Der p 10	0	0	0	0	0	0	0
V Der p 11	3.96	0	0	0	2.62	0	0
V Der p 14	12.45	8.34	4.00	2.87	5.72	0	0
V Der p 15	6.58	2.28	1.71	0	5.16	0	0
V Der p 18	0	0	0	0	0	0	0
V Der p 21	4.81	2.04	1.16	0	0	0	0
V Der p 23	34.72	21.39	11.61	7.54	2.66	0	0
V clone 16	0	0	0	0	6.52	0	0

Donor 2	PI 1:50	PI 1:100	PI 1:200	PI 1:400	M	PI	M
Method	IgG					IgE	
Equ c 1	0	0	0	0	0	0	0
Equ c 3	0	0	0	0	0	0	0
Fag e 2	0	0	0	0	0	0	0
Fel d 1	0	0	0	0	0	0	0
Fel d 2	0	0	0	0	0	0	0
Fel d 4	0	0	0	0	0	0	0
Gad c 1	0	0	0	0	0	0	0
Gal d 1	19.68	9.53	4.04	2.96	3.02	0	0
Gal d 2	8.16	3.48	1.70	1.35	0	0	0
Gal d 3	0	0	0	0	0	0	0
Gal d 5	0	0	0	0	2.98	0	0
Gly m 4	0	0	0	0	0	0	0
Gly m 5	0	0	0	0	0	0	0
Gly m 6	0	0	0	0	0	0	0
Hev b 1	0	0	0	0	4.99	0	0
Hev b 3	0	0	0	0	3.01	0	0
Hev b 5	0	0	0	0	0	0	0
Hev b 6.01	0	0	0	2.11	4.99	0	0
Hev b 8	0	0	0	0	0	0	0
Jug r 1	0	0	0	0	0	0	0
Jug r 2	3.75	2.52	2.15	1.83	0	0	0
Jug r 3	6.22	3.16	1.90	0	0	0	0
Lep d 2	0	0	0	0	0	0	0
Mal d 1	7.30	2.73	0	0	0.83	0	0
Mer a 1	0	0	0	0	0	0	0
Mus m 1	0	0	0	0	0	0	0
MUXF3	0	0	0	0	0	0	0
Ole e 1	0	0	0	0	0	0	0
Ole e 7	0	0	0	0	0	0	0
Ole e 9	39.70	17.86	7.92	3.92	2.52	0	0
Par j 2	41.29	8.95	7.41	4.55	6.12	0	0
Pen m 1	0	0	0	0	0	0	0
Pen m 2	0	0	0	0	0	0	0
Pen m 4	0	0	0	0	0	0	0
Phl p 1	10.08	3.85	3.13	2.00	1.50	0	0
Phl p 2	0	0	0	0	0	0	0
Phl p 4	8.80	4.76	1.89	1.94	0	0.28	0
Phl p 5	0	0	0	0	0	0	0
Phl p 6	0	0	0	0	0	0	0
Phl p 7	0	0	0	0	0	0	0
Phl p 11	0	0	0	0	0	0	0
Phl p 12	0	0	0	0	0	0	0
V Pis v 3	4.47	1.99	0	0	0	0	0
Pla a 1	0	0	0	0	0	0	0
Pla a 2	4.58	1.81	0	0	0	0.20	0
Pla a 3	5.63	0	0	0	0	0	0
Pla l 1	0	0	0	0	0	0	0
Pol d 5	7.09	4.90	0	3.32	0	0.30	0
Pru p 1	0	5.30	0	2.36	0	0	0
Pru p 3	39.56	29.54	16.04	13.13	6.69	0	0
V Pru du 3	0	0	0	0	0	0	0
V Pru du 4	0	0	0	0	0	0	0
V Pru du 6	57.26	39.61	16.73	9.53	4.00	0	0
V Pru du 6.01	0	0	0	0	0	0	0
V Pru du 6.02	3.74	1.94	0	0	0	0	0
Sal k 1	0	0	0	0	0	0	0
Ses i 1	3.38	0	0	1.82	0	0	0
Tri a 14	0	0	0	0	0	0	0
Tri a 19.0101	0	0	0	0	0	0	0
Tri a aA_TI	34.88	18.82	10.32	5.74	2.77	0	0
V Tri a 36 191	2.98	0	0	0	0	0	0
V Tri a 36	3.57	0	0	0	0	0	0
V m43	0	0	0	0	0	0	0
V m82	0	0	0	0	0	0	0
Serine	0	0	0	0	0	0	0
Thioredoxin	0	0	0	0	0	0	0
Glutathione	0	0	0	0	0	0	0
peroxiredoxin	0	0	0	0	0	0	0
Profilin	14.51	8.56	3.78	2.77	0.91	0	0
Dehydrin	0	0	0	0	0	0	0
purothionin	4.05	2.54	0	0	0	0	0
LTP	0	0	0	0	0	0	0
V Ves v 1	0	0	0	0	0	0	0
Ves v 5	0	6.83	3.21	2.26	0	0.29	0
V Ves v 5	0	0	0	0	0	0	0

Donor 3	PI 1:50	PI 1:100	PI 1:200	PI 1:400	M	PI	M
Method	IgG					IgE	
Act d 2	0	0	0	0	0	0	0
Act d 5	0	0	0	0	0	0	0
Act d 8	0	0	0	0	0	0	0
Aln g 1	0	0	0	0	0	0	0
Alt a 1	0	0	0	0	0	0	0
Alt a 6	31.52	19.54	10.77	6.32	0	0.27	0
Amb a 1	0	0	0	0	0	0	0
V Ana o 1	0	0	0	0	2.17	0	0
Ana o 2	0	0	0	0	0	0	0
V Ana o 2	2.39	0	0	0	0	0	0
V nAna o 2	1.70	0	0	0	0	0	0
V Ana o 3	2.28	0	0	0	0	0	0
Ani s 3	0	0	0	0	0	0	0
Api m 1	3.20	3.12	0	0	0	0.31	0
Api m 4	0	0	0	0	0	0	0
Ara h 1	0	0	0	0	0	0.28	0
Ara h 2	0	0	0	0	0	0	0
Ara h 3	0	0	0	0	0	0	0
Ara h 6	0	0	0	0	0	0	0
Ara h 8	0	0	0	0	0	0	0
Ara h 9	0	0	0	0	0	0	0
Art v 1	0	0	0	0	0	0	0
Art v 3	0	0	0	0	0	0	0
Asp f 1	0	0	0	0	0	0	0
Asp f 3	18.70	7.93	4.48	1.70	0	0	0
Asp f 6	0	0	0	0	0	0	0
Ber e 1	0	0	0	0	0	0	0
Bet v 1	2.91	1.94	0	0	0	0	0
Bet v 2	0	0	0	0	0	0	0
Bet v 4	0	0	0	0	0	0	0
Bla g 1	4.69	10.34	4.56	3.86	0	0	0
Bla g 2	13.60	13.24	13.14	12.41	3.68	0	0
Bla g 5	0	0	0	0	0	0	0
Bla g 7	0	0	0	0	0	0	0
Blo t 5	0	0	0	0	0	0	0
Bos d 4	0	0	0	0	0	0	0
V Bos d 4	2.11	0	0	0	0	0	0
Bos d 5	32.48	12.45	4.67	2.19	0	0	0
V Bos d 5	111.41	59.91	32.15	18.45	0	0.29	0
Bos d Lactoferrin	7.16	0	1.85	0	0	0	0
Bos d 8	0	0	0	0	0	0	0
V Bos d 8	21.15	11.47	4.28	2.70	0	0	0
aS1-casein	5.43	0	0	0	0	0	0
aS2-casein	3.50	2.88	0	0	0	0	0
b-casein	0	0	0	0	0	0	0
K-casein	10.22	6.61	2.26	1.39	0	0	0
Transferrin	11.47	7.38	2.85	1.48	0	0	0
Bos d 6	9.07	4.39	0	0	0	0	0
V BSA	5.56	2.98	0	0	0	0.26	0
Can f 1	0	0	0	0	0	0	0
Can f 2	0	0	0	0	0	0	0
Can f 3	5.59	2.5	0	0	0	0	0
V Can f 4	0	0	0	0	0	0	0
Can f 5	0	0	0	0	0	0	0
V Can f 5	0	0	0	0	0	0	0
V Can f 6	0	0	0	0	0	0	0
Che a 1	0	0	0	0	0	0	0
Cla h 8	7.68	0	0	0	0	0	0
Cor a 1.0401	0	0	0	0	0	0	0
Cor a 8	19.64	13.38	9.38	4.56	0	0	0
Cor a 9	0	0	0	0	0	0	0
Cry j 1	1.46	0	0	0	0	0.27	0
Cyn d 1	0	0	0	0	0	0	0
Cup a 1	4.00	2.21	0	0	0	0	0
Der f 1	2.08	0	0	0	0	0	0
Der f 2	0	0	0	0	0	0	0
Der p 1	0	0	0	0	0	0	0
Der p 2	0	0	0	0	0	0	0
V Der p 4	0	0	0	0	0	0	0
V Der p 5	4.58	0	0	0	0	0	0
V Der p 7	0	0	0	0	0	0	0
Der p 10	0	0	0	0	0	0	0
V Der p 11	0	0	0	0	0	0	0
V Der p 14	2.08	0	0	0	0	0	0
V Der p 15	0	0	0	0	0	0	0
V Der p 18	0	0	0	0	0	0	0
V Der p 21	0	0	0	0	0	0	0
V Der p 23	0	0	0	0	0	0	0
V clone 16	2.19	0	0	0	0	0	0

Donor 3	PI 1:50	PI 1:100	PI 1:200	PI 1:400	M	PI	M
Method	IgG					IgE	
Equ c 1	0	0	0	0	0	0	0
Equ c 3	0	0	0	0	0	0	0
Fag e 2	0	0	0	0	0	0	0
Fel d 1	1.96	1.85	0	0	0	0	0
Fel d 2	0	0	0	0	0	0	0
Fel d 4	0	0	0	0	0	0	0
Gad c 1	0	0	0	0	0	0	0
Gal d 1	77.34	29.03	9.14	3.30	1.96	0	0
Gal d 2	64.51	31.21	20.04	10.78	3.90	0	0
Gal d 3	2.77	0	0	0	0	0	0
Gal d 5	0	0	0	0	0	0	0
Gly m 4	0	0	0	0	0	0	0
Gly m 5	0	0	0	0	0	0	0
Gly m 6	0	0	0	0	0	0	0
Hev b 1	3.19	2.61	0	0	0	0	0
Hev b 3	0	0	0	0	0	0	0
Hev b 5	0	0	0	0	0	0	0
Hev b 6.01	6.07	3.54	0	0	1.31	0	0
Hev b 8	0	0	0	0	0	0	0
Jug r 1	0	0	0	0	0	0	0
Jug r 2	0	0	4.11	5.12	0	0	0
Jug r 3	0	0	0	0	0	0	0
Lep d 2	0	0	0	0	0	0	0
Mal d 1	20.71	1.85	0	0	1.82	0	0
Mer a 1	0	0	0	0	0	0	0
Mus m 1	0	0	0	0	0	0	0
MUXF3	0	0	0	0	0	0	0
Ole e 1	0	0	0	0	0	0	0
Ole e 7	0	0	0	0	0	0	0
Ole e 9	97.54	47.00	30.39	22.12	4.73	0	0
Par j 2	37.07	8.05	6.26	0	3.36	0	0
Pen m 1	0	0	0	0	0	0	0
Pen m 2	8.08	3.39	1.54	0	0	0	0
Pen m 4	0	0	0	0	0	0	0
Phl p 1	7.53	3.96	1.82	0	0	0.29	0
Phl p 2	0	0	0	0	0	0	0
Phl p 4	0	0	0	0	0	0	0
Phl p 5	0	0	0	0	0	0	0.30
Phl p 6	0	0	0	0	0	0	0
Phl p 7	0	0	0	0	1.70	0	0
Phl p 11	0	0	0	0	0	0	0
Phl p 12	0	0	0	0	0	0	0
V Pis v 3	0	0	0	0	0	0	0
Pla a 1	0	0	0	0	0	0	0
Pla a 2	0	0	0	0	0	0	0
Pla a 3	0	0	0	0	0	0	0
Pla l 1	0	0	0	0	0	0	0
Pol d 5	0	4.33	0	0	0	0	0
Pru p 1	0	0	0	0	0	0	0
Pru p 3	41.96	39.51	19.98	15.05	2.52	0	0
V Pru du 3	0	0	0	0	0	0	0
V Pru du 4	5.60	0	0	0	0	0	0
V Pru du 6	0	0	0	0	0	0	0
V Pru du 6.01	0	0	0	0	0	0	0
V Pru du 6.02	0	0	0	0	0	0	0
Sal k 1	183.27	150.07	120.39	79.79	30.46	0	0
Ses i 1	2.65	4.30	2.88	2.65	0	0	0
Tri a 14	0	0	0	0	0	0	0
Tri a 19.0101	0	0	0	0	0	0	0
Tri a aA_TI	8.30	0	3.19	0	0	0	0
V Tri a 36 191	2.03	0	0	0	0	0	0
V Tri a 36	0	0	0	0	0	0	0
V m43	3.10	0	0	0	0	0	0
V m82	0	0	0	0	0	0	0
Serine	0	0	0	0	4.32	0	0
Thioredoxin	0	0	0	0	0	0	0
Glutathione	0	0	0	0	0	0	0
peroxiredoxin	0	0	0	0	0	0	0
Profilin	0	0	0	0	5.09	0	0
Dehydrin	0	0	0	0	0	0	0
purothionin	0	0	0	0	0	0	0
LTP	0	0	0	0	0	0	0
V Ves v 1	0	0	0	0	0	0	0
Ves v 5	0	0	0	0	0	0	0
V Ves v 5	0	0	0	0	0	0	0

Donor 4	PI 1:50	PI 1:100	PI 1:200	PI 1:400	M	PI	M
Method	IgG					IgE	
Act d 2	0	0	0	0	0	0	0
Act d 5	0	0	0	0	0	0	0
Act d 8	0	0	0	0	0	0	0
Aln g 1	0	0	0	0	0	0	0
Alt a 1	0	0	0	0	0	0	0
Alt a 6	3.78	1.78	1.72	0	0	0	0
Amb a 1	0	0	0	0	0	0	0
V Ana o 1	0	0	0	0	0	0	0
Ana o 2	3.92	0	0	0	0	0	0
V Ana o 2	3.41	0	0	0	0	0	0
V nAna o 2	61.56	55.11	28.10	14.30	4.90	0	0
V Ana o 3	12.41	3.48	7.70	3.35	3.59	0	0
Ani s 3	2.61	0	0	0	0	0	0
Api m 1	0	0	0	0	1.45	0.35	0
Api m 4	0	0	0	0	0	0	0
Ara h 1	1.97	0	5.50	0	0	0.29	0
Ara h 2	0	0	0	0	0	0	0
Ara h 3	0	0	0	0	0	0	0
Ara h 6	0	0	0	0	0	0	0
Ara h 8	0	0	0	0	0	0	0
Ara h 9	0	0	0	0	0	0	0
Art v 1	0	0	0	0	0	0	0
Art v 3	0	0	0	0	0	0	0
Asp f 1	0	0	0	0	0	0	0
Asp f 3	0	0	0	0	0	0	0
Asp f 6	0	0	0	0	0	0	0
Ber e 1	0	0	0	0	0	0	0
Bet v 1	0	0	0	0	0	0	0
Bet v 2	0	0	0	0	0	0	0
Bet v 4	0	0	0	0	0	0	0
Bla g 1	2.65	1.53	0	0	0	0	0
Bla g 2	8.41	9.32	2.47	1.38	3.72	0	0
Bla g 5	10.29	6.15	4.23	1.72	0	0	0
Bla g 7	2.44	0	0	0	0	0	0
Blo t 5	0	0	0	0	0	0	0
Bos d 4	52.17	20.91	21.50	12.07	0	0	0
V Bos d 4	98.89	56.06	41.78	21.21	3.03	0.30	0
Bos d 5	23.43	21.43	35.87	8.12	0	0	0
V Bos d 5	179.00	149.65	148.61	91.44	1.48	0.37	0
Bos d Lactoferrin	37.54	20.06	28.15	8.47	5.42	0	0
Bos d 8	9.32	0	7.76	4.45	0	0	0
V Bos d 8	176.54	135.52	81.76	43.36	20.04	0.30	0
aS1-casein	28.49	2.9	22.17	5.34	6.38	0	0
aS2-casein	1.46	1.48	0	0	0	0	0
b-casein	0	0	0	0	4.32	0	0
K-casein	2.72	20.98	20.99	15.54	7.58	0	0
Transferrin	67.89	18.05	33.08	15.35	4.52	0.27	0
Bos d 6	138.68	79.24	46.98	20.15	18.73	0.32	0
V BSA	125.33	66.4	40.93	16.96	21.53	0.30	0
Can f 1	0	0	0	0	0	0	0
Can f 2	0	0	0	0	0	0	0
Can f 3	5.87	3.13	0	0	3.27	0	0
V Can f 4	0	0	0	0	0	0	0
Can f 5	0	0	0	0	0	0	0
V Can f 5	0	0	0	0	0	0	0
V Can f 6	0	0	0	0	0	0	0
Che a 1	0	0	0	0	0	0	0
Cla h 8	0	0	3.39	0	0	0	0
Cor a 1.0401	0	0	0	0	0	0	0
Cor a 8	8.29	2.69	7.78	0	3.81	0	0
Cor a 9	28.89	18.31	14.19	5.60	0	0	0
Cry j 1	9.31	3.90	3.60	1.50	0	0	0
Cyn d 1	28.13	14.44	12.44	4.14	2.19	0	0
Cup a 1	8.21	3.56	3.82	1.09	0	0	0
Der f 1	7.39	4.04	7.60	3.85	0	0	0
Der f 2	0	0	0	0	0	0	0
Der p 1	0	0	1.93	0	0	0	0
Der p 2	0	0	0	0	0	0	0
V Der p 4	0	0	0	0	0	0	0
V Der p 5	0	0	0	0	0	0	0
V Der p 7	2.59	0	0	0	0	0	0
Der p 10	0	0	0	0	0	0	0
V Der p 11	4.47	4.83	0	0	2.34	0	0
V Der p 14	15.33	10.47	4.50	0	2.88	0	0
V Der p 15	0	1.30	0	0	3.14	0	0
V Der p 18	3.01	0	0	0	0	0	0
V Der p 21	5.36	2.45	0	0	0	0	0
V Der p 23	20.06	9.96	23.22	6.95	3.82	0	0
V clone 16	0	0	0	0	0	0	0

Donor 4	PI 1:50	PI 1:100	PI 1:200	PI 1:400	M	PI	M
Method	IgG					IgE	
Equ c 1	0	0	0	0	0	0	0
Equ c 3	0	0	0	0	0	0	0
Fag e 2	0	0	0	0	0	0	0
Fel d 1	5.34	4.43	3.30	1.53	2.25	0	0
Fel d 2	2.59	1.89	3.74	0	2.10	0	0
Fel d 4	0	0	0	0	0	0	0
Gad c 1	0	0	0	0	0	0	0
Gal d 1	105.84	48.87	30.79	13.64	10.91	0	0
Gal d 2	45.88	32.88	33.29	20.01	6.45	0	0
Gal d 3	0	0	0	0	2.69	0	0
Gal d 5	0	1.52	0	0	12.30	0.36	0
Gly m 4	0	0	0	0	0	0	0
Gly m 5	0	0	0	0	0	0	0
Gly m 6	2.83	1.81	0	0	0	0	0
Hev b 1	21.44	9.89	9.02	4.10	3.43	0	0
Hev b 3	26.85	16.41	7.98	3.70	3.75	0.25	0
Hev b 5	0	0	0	0	0	0	0
Hev b 6.01	31.62	12.91	9.78	3.10	4.22	0.29	0
Hev b 8	22.37	11.82	7.56	1.59	0	0	0
Jug r 1	7.32	2.45	0	0	0	0	0
Jug r 2	15.06	7.13	3.08	1.74	4.03	0	0
Jug r 3	3.65	0	2.61	1.34	0	0	0
Lep d 2	0	0	0	0	0	0	0
Mal d 1	1.60	7.67	10.88	0	0	0.27	0
Mer a 1	0	0	0	0	0	0	0
Mus m 1	0	0	0	0	0	0	0
MUXF3	0	0	0	0	0	0	0
Ole e 1	0	0	0	0	0	0	0
Ole e 7	0	0	0	0	0	0	0
Ole e 9	82.19	42.81	31.35	12.33	4.69	0	0
Par j 2	10.07	3.24	27.13	9.38	4.61	0	0
Pen m 1	3.30	0	0	0	0	0	0
Pen m 2	0	0	0	0	0	0	0
Pen m 4	0	0	0	0	0	0	0
Phl p 1	11.38	6.70	6.23	3.52	2.99	0.28	0
Phl p 2	0	0	0	0	0	0	0
Phl p 4	35.37	18.30	12.71	5.93	2.10	0	0
Phl p 5	83.68	37.55	40.47	16.66	4.04	0	0
Phl p 6	0	0	0	0	0	0	0
Phl p 7	0	0	0	0	0	0	0
Phl p 11	0	0	0	0	0	0	0
Phl p 12	0	0	0	0	0	0	0
V Pis v 3	0	0	0	0	2.07	0	0
Pla a 1	0	0	0	0	0	0	0
Pla a 2	12.09	5.28	5.02	0	0	0	0
Pla a 3	0	0	0	0	0	0	0
Pla l 1	0	0	0	0	0	0	0
Pol d 5	2.17	1.10	0	0	0	0	0
Pru p 1	0	0	0	0	0	0	0
Pru p 3	84.98	48.89	33.4	18.74	7.10	0	0
V Pru du 3	0	0	0	0	0	0	0
V Pru du 4	2.28	0	0	0	0	0	0
V Pru du 6	27.82	13.43	9.72	3.85	0	0	0
V Pru du 6.01	5.86	4.10	0	0	0	0	0
V Pru du 6.02	4.07	2.30	0	0	0	0	0
Sal k 1	0	0	0	0	0	0.27	0
Ses i 1	0	0	2.39	0	0	0	0
Tri a 14	59.34	31.10	28.30	14.40	4.61	0	0
Tri a 19.0101	0	0	4.23	0	0	0	0
Tri a aA_TI	164.08	134.51	94.46	55.35	11.39	0	0
V Tri a 36 191	9.42	2.79	3.79	0	0	0	0
V Tri a 36	0	4.59	4.72	0	0	0	0
V m43	5.49	3.63	4.51	1.34	1.60	0	0
V m82	0	0	0	0	0	0	0
Serine	0	0	0	0	0	0	0
Thioredoxin	0	1.57	0	0	0	0	0
Glutathione	4.63	0	0	0	0	0	0
peroxiredoxin	0	0	0	0	0	0	0
Profilin	0	1.90	0	0	2.25	0	0
Dehydrin	0	0	0	0	0	0	0
purothionin	9.47	7.24	5.07	2.45	1.78	0	0
LTP	1.53	0	0	0	0	0	0
V Ves v 1	0	0	0	0	0	0	0
Ves v 5	2.01	0	0	0	0	0	0
V Ves v 5	0	1.35	0	0	0	0	0
