# Supplementary material for: Transmission of allergen-specific IgG and IgE from maternal blood into breast milk visualized with microarray technology
Source: J Allergy Clin Immunol. 2014 Nov;134(5):1213–5. doi: 10.1016/j.jaci.2014.08.041 (PMC4220005; doi:10.1016/j.jaci.2014.08.041)
Supplement: Supplementary Figure E2 [file mmc3.pdf]

Supplementary Figure E2

Sensitized mothers (Donors 5-23)

Patient	Donor 5		Donor 6		Donor 7		Donor 8		Donor 9		Donor 10	
Sample	Pl	M	Pl	M	Pl	M	Pl	M	Pl	M	Pl	M
Method	IgE		IgE		IgE		IgE		IgE		IgE	
Act d 2	0	0	0	0	0	0	0	0	0	0	0	0
Act d 5	0	0	0	0	0	0	0	0	0	0	0	0
Act d 8	0	0	0	0	0	0	0	0	0	0	0	0
Aln g 1	0	0	0	0	0	0	0.25	0	8.39	0	0	0
Alt a 1	0	0	0	0	0	0	0	0	0	0	0	0
Alt a 6	0	0	0	0	0	0	0	0	0	0	0	0
Amb a 1	0	0	0	0	0	0	0	0	0	0	0	0
V Ana o 1	0	0	0	0	0	0	0	0	0	0	0	0
Ana o 2	0	0	0	0	0	0	0	0	0	0	0	0
V Ana o 2	0	0	0	0	0	0	0	0	0	0	0	0
V nAna o 2	0	0	0	0	0	0	0	0	0	0	0	0
V Ana o 3	0	0	0	0	0	0	0	0	0	0	0	0
Ani s 3	0	0	0	0	0	0	0	0	0	0	0	0
Api m 1	0	0	0	0	0	0	0	0	0	0	0	0
Api m 4	0	0	0	0	0	0	0	0	0	0	0	0
Ara h 1	0	0	0	0	0	0	0	0	0	0	0	0
Ara h 2	0	0	0	0	0	0	0	0	0	0	0	0
Ara h 3	0	0	0	0	0	0	0	0	0	0	0	0
Ara h 6	0	0	0	0	0	0	0	0	0	0	0	0
Ara h 8	0	0	0	0	0	0	0.19	0	11.45	0	0	0
Ara h 9	0	0	0	0	0	0	0	0	0	0	0	0
Art v 1	0	0	2.66	0	0	0	0	0	0	0	3.41	0
Art v 3	0	0	0	0	0	0	0	0	0	0	0	0
Asp f 1	0	0	0	0	0	0	0	0	0	0	0	0
Asp f 3	0	0	0	0	0	0	0	0	0	0	0	0
Asp f 6	0	0	0	0	0	0	0	0	0	0	0	0
Ber e 1	0	0	0	0	0	0	0	0	0	0	0	0
Bet v 1	0	0	0	0	0	0	5.21	0	71.92	0.19	0	0
Bet v 2	0	0	0	0	0	0	0	0	0	0	0	0
Bet v 4	0	0	0	0	0	0	0	0	0	0	0	0
Bla g 1	0	0	0	0	0	0	0	0	0	0	0	0
Bla g 2	0	0	0	0	0	0	0	0	0	0	0	0
Bla g 5	0	0	0	0	0	0	0	0	0	0	0	0
Bla g 7	0	0	0	0	0	0	0	0	0	0	0	0
Blo t 5	0	0	0	0	0	0	0	0	0	0	0	0
Bos d 4	0	0	0	0	0	0	0	0	0	0	0	0
V Bos d 4	0	0	0	0	0	0	0	0	0	0	0	0
Bos d 5	0	0	0	0	0	0	0	0	0	0	0	0
V Bos d 5	0	0	0	0	0	0	0	0	0	0	0	0
Bos d Lactoferrin	0	0	0	0	0	0	0	0	0	0	0	0
Bos d 8	0	0	0	0	0	0	0	0	0	0	0	0
V Bos d 8	0	0	0	0	0	0	0	0	0	0	0	0
aS1-casein	0	0	0	0	0	0	0	0	0	0	0	0
aS2-casein	0	0	0	0	0	0	0	0	0	0	0	0
b-casein	0	0	0	0	0	0	0	0	0	0	0	0
K-casein	0	0	0	0	0	0	0	0	0	0	0	0
Transferrin	0	0	0	0	0	0	0	0	0	0	0	0
Bos d 6	0	0	0	0	0	0	0	0	0	0	0	0
V BSA	0	0	0	0	0	0	0	0	0	0	0	0
Can f 1	0	0	0	0	0	0	19.78	0	0	0	0	0
Can f 2	0	0	0	0	0	0	8.10	0	0	0	0	0
Can f 3	0	0	0	0	0	0	0	0	0	0	0	0
V Can f 4	0	0	0	0	0	0	0	0	0	0	0	0
Can f 5	0	0	0	0	0	0	11.17	0	0	0	0.18	0
V Can f 5	0	0	0	0	0	0	0.24	0	0	0	0	0
V Can f 6	0	0	0	0	0	0	4.54	0	0	0	0	0
Che a 1	0	0	0	0	0	0	0	0	0	0	0	0
Cla h 8	0	0	0	0	0	0	0	0	0	0	0	0
Cor a 1.0401	0	0	0	0	0	0	0.21	0	24.08	0	0	0
Cor a 8	0	0	0	0	0	0	0	0	0	0	0	0
Cor a 9	0	0	0	0	0	0	0	0	0	0	0	0
Cry j 1	0	0	0	0	0	0	0	0	0	0	0	0
Cyn d 1	0	0	5.08	0	45.82	0	1.11	0	0.64	0	1.98	0
Cup a 1	0	0	0	0	0	0	0	0	0	0	0	0
Der f 1	0	0	0	0	0	0	0	0	0	0	0	0
Der f 2	0.40	0	0	0	0	0	0	0	0	0	0	0
Der p 1	0	0	0	0	0	0	0	0	0	0	0	0
Der p 2	0.44	0	0	0	0	0	0	0	0	0	0	0
V Der p 4	0	0	0	0	0	0	0	0	0	0	0	0
V Der p 5	0	0	0	0	0	0	0	0	0	0	0	0
V Der p 7	0	0	0	0	0	0	0	0	0	0	0	0
Der p 10	0	0	0	0	0	0	0	0	0	0	0	0
V Der p 11	0	0	0	0	0	0	0	0	0	0	0	0
V Der p 14	0	0	0	0	0	0	0	0	0	0	0	0
V Der p 15	0	0	0	0	0	0	0	0	0	0	0	0
V Der p 18	0	0	0	0	0	0	0	0	0	0	0	0
V Der p 21	0	0	0	0	0	0	0	0	0	0	0	0
V Der p 23	0.62	0	0	0	0	0	0	0	0	0	0	0
V clone 16	0	0	0	0	0	0	0	0	0	0	0	0

Patient	Donor 5		Donor 6		Donor 7		Donor 8		Donor 9		Donor 10	
Sample	PI	M	PI	M	PI	M	PI	M	PI	M	PI	M
Method	IgE		IgE		IgE		IgE		IgE		IgE	
Equ c 1	0	0	0	0	0	0	31.07	0	0	0	0	0
Equ c 3	0	0	0	0	0	0	0	0	0	0	0	0
Fag e 2	0	0	0	0	0	0	0	0	0	0	0	0
Fel d 1	13.28	0	0	0	0	0	1.44	0	0	0	0	0
Fel d 2	0	0	0	0	0	0	0	0	0	0	0	0
Fel d 4	0	0	0	0	0	0	1.59	0	0	0	0	0
Gad c 1	0	0	0	0	0	0	0	0	0	0	0	0
Gal d 1	0	0	0	0	0	0	0	0	0	0	0	0
Gal d 2	0	0	0	0	0	0	0	0	0	0	0	0
Gal d 3	0	0	0	0	0	0	0	0	0	0	0	0
Gal d 5	0	0	0	0	0	0	0	0	0	0	0	0
Gly m 4	0	0	0	0	0	0	0	0	0.91	0	0	0
Gly m 5	0	0	0	0	0	0	0	0	0	0	0	0
Gly m 6	0	0	0	0	0	0	0	0	0	0	0	0
Hev b 1	0	0	0	0	0	0	0	0	0	0	0	0
Hev b 3	0	0	0	0	0	0	0	0	0	0	0	0
Hev b 5	0	0	0	0	0	0	0	0	0	0	0	0
Hev b 6.01	0	0	0	0	0	0	0	0	0	0	0	0
Hev b 8	0	0	0	0	0	0	0	0	0	0	0	0
Jug r 1	0	0	0	0	0	0	0	0	0	0	0	0
Jug r 2	0	0	0	0	0.11	0	0	0	0	0	0	0
Jug r 3	0	0	0	0	0	0	0	0	0	0	0	0
Lep d 2	0	0	0	0	0	0	0	0	0	0	0	0
Mal d 1	0	0	0	0	0	0	0.82	0	1.06	0	0	0
Mer a 1	0	0	0	0	0	0	0	0	0	0	0	0
Mus m 1	0	0	0	0	0	0	1.96	0	0	0	0	0
MUXF3	0	0	0	0	0	0	0	0	0	0	0	0
Ole e 1	0.43	0	0	0	0	0	0	0	0	0	0	0
Ole e 7	0	0	0	0	0	0	0	0	0	0	0	0
Ole e 9	0	0	0	0	0	0	0	0	0	0	0	0
Par j 2	0	0	0	0	0	0	0	0	0	0	0	0
Pen m 1	0	0	0	0	0	0	0	0	0	0	0	0
Pen m 2	0	0	0	0	0.19	0	0	0	0	0	0	0
Pen m 4	0	0	0	0	0	0	0	0	0	0	0	0
Phl p 1	0.23	0	46.22	0	58.75	0	7.93	0	10.00	0	7.10	0
Phl p 2	0	0	0	0	0	0	2.80	0	0	0	0	0
Phl p 4	0	0	0.18	0	10.67	0	2.39	0	0	0	0	0
Phl p 5	0	0	29.97	0	8.87	0	12.00	0	0	0	0	0
Phl p 6	0	0	13.75	0	8.02	0	0.42	0	0	0	0	0
Phl p 7	0	0	0.17	0	6.44	0	0	0	0	0	0	0
Phl p 11	0	0	0	0	0	0	0	0	0	0	0	0
Phl p 12	0	0	0	0	0	0	0	0	0	0	0	0
V Pis v 3	0	0	0	0	0	0	0	0	0	0	0	0
Pla a 1	0	0	0	0	0	0	0	0	0	0	0	0
Pla a 2	0	0	0	0	0.13	0	0	0	0	0	0	0
Pla a 3	0	0	0	0	0	0	0	0	0	0	0	0
Pla l 1	0	0	0	0	0	0	0	0	0	0	0	0
Pol d 5	0	0	1.88	0	0.11	0	0	0	0	0	0	0
Pru p 1	0	0	0	0	0	0	0	0	3.08	0	0	0
Pru p 3	0	0	0	0	0	0	0	0	0	0	0	0
V Pru du 3	0	0	0	0	0	0	0	0	0	0	0	0
V Pru du 4	0	0	0	0	0	0	0	0	0	0	0	0
V Pru du 6	0	0	0	0	0	0	0	0	0	0	0	0
V Pru du 6.01	0	0	0	0	0	0	0	0	0	0	0	0
V Pru du 6.02	0	0	0	0	0	0	0	0	0	0	0	0
Sal k 1	0	0	0	0	0	0	0	0	0	0	0	0
Ses i 1	0	0	0	0	0	0	0	0	0	0	0	0
Tri a 14	0	0	0	0	0	0	0	0	0	0	0	0
Tri a 19.0101	0	0	0	0	0	0	0	0	0	0	0	0
Tri a aA_TI	0	0	0	0	0	0	0	0	0	0	0	0
V Tri a 36 191	0	0	0	0	0	0	0	0	0	0	0	0
V Tri a 36	0	0	0	0	0	0	0	0	0	0	0	0
V m43	0	0	0	0	0	0	0	0	0	0	0	0
V m82	0	0	0	0	0	0	0	0	0	0	0	0
Serine	0	0	0	0	0	0	0	0	0	0	0	0
Thioredoxin	0	0	0	0	0	0	0	0	0	0	0	0
Glutathione	0	0	0	0	0	0	0	0	0	0	0	0
peroxiredoxin	0	0	0	0	0	0	0	0	0	0	0	0
Profilin	0	0	0	0	0	0	0	0	0	0	0	0
Dehydrin	0	0	0	0	0	0	0	0	0	0	0	0
purothionin	0	0	0	0	0	0	0	0	0	0	0	0
LTP	0	0	0	0	0	0	0	0	0	0	0	0
V Ves v 1	0	0	0	0	0	0	0	0	0	0	0	0
Ves v 5	0	0	2.58	0	0	0	0	0	0.16	0	0	0
V Ves v 5	0	0	0	0	0	0	0	0	0	0	0	0

IgE	
0.1-0.99	(yellow)
1-14.99	(orange)
≥ 15	(red)

Patient	Donor 11		Donor 12		Donor 13		Donor 14		Donor 15		Donor 16	
Sample	PI	M	PI	M	PI	M	PI	M	PI	M	PI	M
Method	IgE		IgE		IgE		IgE		IgE		IgE	
Act d 2	0	0	0	0	0	0	0	0	0	0	0	0
Act d 5	0	0	0	0	0	0	0	0	0	0	0	0
Act d 8	0	0	0	0	0	0	0	0	0	0	0	0
Aln g 1	0	0	0.11	0	16.73	0	0.81	0	10.38	0	1.53	0
Alt a 1	0	0	0	0	0	0	0.57	0	0	0	0	0
Alt a 6	0	0	0	0	0	0	0	0	0	0	0	0
Amb a 1	0	0	0	0	3.52	0	0	0	0	0	0	0
V Ana o 1	0	0	0	0	0	0	0	0	0	0	0	0
Ana o 2	0	0	0	0	0	0	0	0	0	0	0	0
V Ana o 2	0	0	0	0	0	0	0	0	0	0	0	0
V nAna o 2	0	0	0	0	0	0	0	0	0	0	0	0
V Ana o 3	0	0	0	0	0	0	0	0	0	0	0	0
Ani s 3	0	0	0	0	0	0	0	0	0	0	0	0
Api m 1	0	0	0	0	0.61	0	0	0	0	0	0	0
Api m 4	0	0	0	0	0	0	0	0	0	0	0	0
Ara h 1	0	0	0	0	0	0	0	0	0	0	0	0
Ara h 2	0	0	0	0	0	0	0	0	0	0	0	0
Ara h 3	0	0	0	0	0	0	0	0	0	0	0	0
Ara h 6	0	0	0	0	0	0	0	0	0	0	0	0
Ara h 8	0	0	0	0	18.15	0	0.13	0	1.77	0	0.41	0
Ara h 9	0	0	0	0	0	0	0	0	0	0	0	0
Art v 1	0	0	0.26	0	0	0	0	0	0	0	0	0
Art v 3	0	0	0	0	0	0	0	0	0	0	0	0
Asp f 1	0	0	0	0	0	0	0	0	0	0	0	0
Asp f 3	0	0	0	0	0	0	0	0	0	0	0	0
Asp f 6	0	0	0	0	0	0	0	0	0	0	0	0
Ber e 1	0	0	0	0	0	0	0	0	0	0	0	0
Bet v 1	0.64	0	1.90	0	83.41	0.15	11.28	0	68.86	0.11	5.56	0
Bet v 2	0	0	0	0	0	0	0	0	0	0	0	0
Bet v 4	0	0	0	0	0	0	0	0	0	0	0	0
Bla g 1	0	0	0	0	0	0	0	0	0	0	0	0
Bla g 2	0	0	0	0	0	0	0	0	0	0	0	0
Bla g 5	0	0	0	0	0	0	0	0	0	0	0	0
Bla g 7	0	0	0	0	0	0	0	0	0	0	0	0
Blo t 5	0	0	0	0	0	0	0	0	0	0	0	0
Bos d 4	0	0	0	0	0	0	0	0	0	0	0	0
V Bos d 4	0	0	0	0	0	0	0	0	0	0	0	0
Bos d 5	0	0	0	0	0	0	0	0	0	0	0	0
V Bos d 5	0	0	0	0	0	0	0	0	0	0	0	0
Bos d Lactoferrin	0	0	0	0	0	0	0	0	0	0	0	0
Bos d 8	0	0	0	0	0	0	0	0	0	0	0	0
V Bos d 8	0	0	0	0	0	0	0	0	0	0	0	0
aS1-casein	0	0	0	0	0	0	0	0	0	0	0	0
aS2-casein	0	0	0	0	0	0	0	0	0	0	0	0
b-casein	0	0	0	0	0	0	0	0	0	0	0	0
K-casein	0	0	0	0	0	0	0	0	0	0	0	0
Transferrin	0	0	0	0	0	0	0	0	0	0	0	0
Bos d 6	0	0	0	0	0	0	0	0	0	0	0	0
V BSA	0	0	0	0	0	0	0	0	0	0	0	0
Can f 1	0	0	0.48	0	0	0	0	0	0	0	0	0
Can f 2	0	0	0.81	0	0	0	0	0	0	0	0	0
Can f 3	0	0	0	0	0	0	0	0	0	0	0	0
V Can f 4	0	0	0	0	0	0	0	0	0	0	0	0
Can f 5	0	0	4.18	0	0	0	0	0	0	0	0	0
V Can f 5	0	0	0	0	0	0	0	0	0	0	0	0
V Can f 6	0.14	0	7.86	0	0	0	0	0	0	0	0.31	0
Che a 1	0	0	0	0	0	0	0	0	0	0	0	0
Cla h 8	0	0	0	0	0	0	0	0	0	0	0	0
Cor a 1.0401	0.52	0	0.51	0	22.00	0	0.66	0	9.25	0	1.41	0
Cor a 8	0	0	0	0	0	0	0	0	0	0	0	0
Cor a 9	0	0	0	0	0	0	0	0	0	0	0	0
Cry j 1	0	0	0	0	0	0	0	0	0	0	0	0
Cyn d 1	0	0	0	0	1.72	0	0.72	0	5.51	0	0.73	0
Cup a 1	0	0	0	0	0	0	0	0	0	0	0	0
Der f 1	0	0	0	0	3.06	0	0	0	0	0	0	0
Der f 2	0.57	0	0	0	9.53	0	0	0	0	0	0	0
Der p 1	0	0	0	0	1.83	0	0	0	0	0	0	0
Der p 2	0.78	0	0	0	8.48	0	0	0	0	0	0	0
V Der p 4	0	0	0	0	0	0	0	0	0	0	0	0
V Der p 5	0	0	0	0	0	0	0	0	0	0	0	0
V Der p 7	0	0	0	0	2.44	0	0	0	0	0	0	0
Der p 10	0	0	0	0	0	0	0	0	0	0	0	0
V Der p 11	0	0	0	0	0	0	0	0	0	0	0	0
V Der p 14	0	0	0	0	0	0	0	0	0	0	0	0
V Der p 15	0	0	0	0	0	0	0	0	0	0	0	0
V Der p 18	0	0	0	0	0	0	0	0	0	0	0	0
V Der p 21	0	0	0	0	0	0	0	0	0	0	0	0
V Der p 23	0	0	0	0	0.87	0	0	0	0	0	0	0
V clone 16	0	0	0	0	0	0	0	0	0	0	0	0

Patient	Donor 11		Donor 12		Donor 13		Donor 14		Donor 15		Donor 16	
Sample	PI	M	PI	M	PI	M	PI	M	PI	M	PI	M
Method	IgE		IgE		IgE		IgE		IgE		IgE	
Equ c 1	0	0	1.24	0	0	0	0	0	0	0	32.54	0.14
Equ c 3	0	0	0	0	0	0	0	0	0	0	0.23	0
Fag e 2	0	0	0	0	0	0	0	0	0	0	0	0
Fel d 1	0.29	0	10.90	0	0	0	7.58	0	2.28	0	1.93	0
Fel d 2	0	0	0	0	0	0	0	0	0	0	0	0
Fel d 4	0.39	0	9.60	0	0	0	0	0	0	0	0.54	0
Gad c 1	0	0	0	0	0	0	0	0	0	0	0	0
Gal d 1	0	0	0	0	0	0	0	0	0	0	0	0
Gal d 2	0	0	0	0	0	0	0	0	0	0	0	0
Gal d 3	0	0	0	0	0	0	0	0	0	0	0	0
Gal d 5	0	0	0	0	0	0	0	0	0	0	0	0
Gly m 4	0	0	0	0	12.19	0	0	0	0.14	0	0	0
Gly m 5	0	0	0	0	0	0	0	0	0	0	0	0
Gly m 6	0	0	0	0	0	0	0	0	0	0	0	0
Hev b 1	0	0	0	0	0	0	0	0	0	0	0	0
Hev b 3	0	0	0	0	0	0	0	0	0	0	0	0
Hev b 5	0	0	0	0	0	0	0	0	0	0	0	0
Hev b 6.01	0	0	0	0	0	0	0.10	0	0	0	0	0
Hev b 8	0	0	0	0	0	0	0	0	0	0	0	0
Jug r 1	0	0	0	0	0	0	0	0	0	0	0	0
Jug r 2	0	0	0	0	0.57	0	0	0	0	0	0	0
Jug r 3	0	0	0	0	0	0	0	0	0	0	0	0
Lep d 2	0	0	0	0	0	0	0	0	0	0	0	0
Mal d 1	0.42	0	0.11	0	33.68	0	0.42	0	12.36	0	0.51	0
Mer a 1	0	0	0	0	0	0	0	0	0	0	0	0
Mus m 1	0.26	0	0.60	0	0.54	0	2.06	0	0	0	4.09	0
MUXF3	0	0	0	0	0	0	0	0	0	0	0	0
Ole e 1	0	0	0	0	0	0	0	0	0	0	0	0
Ole e 7	0	0	0	0	0	0	0	0	0	0	0	0
Ole e 9	0	0	0	0	0	0	0	0	0	0	0	0
Par j 2	0	0	0	0	0	0	0	0	0	0	0	0
Pen m 1	0	0	0	0	0	0	0	0	0	0	0	0
Pen m 2	0	0	0	0	0	0	0	0	0	0	0	0
Pen m 4	0	0	0	0	0	0	0	0	0	0	0	0
Phl p 1	0	0	3.95	0	0.52	0	7.41	0	20.39	0	8.11	0
Phl p 2	0	0	0	0	0	0	0.88	0	0	0	0	0
Phl p 4	0.48	0	0	0	1.82	0	0.11	0	0	0	2.99	0
Phl p 5	0	0	0	0	0	0	0.35	0	0	0	0	0
Phl p 6	0	0	0	0	0	0	0	0	0	0	0	0
Phl p 7	0	0	0	0	0	0	0	0	0	0	0	0
Phl p 11	0	0	0	0	0	0	0	0	0	0	0	0
Phl p 12	0	0	0	0	0	0	0	0	0	0	0	0
V Pis v 3	0	0	0	0	0	0	0	0	0	0	0	0
Pla a 1	0	0	0	0	0	0	0	0	0	0	0	0
Pla a 2	0	0	0	0	0.29	0	0	0	0	0	0	0
Pla a 3	0	0	0	0	0	0	0	0	0	0	0	0
Pla l 1	0	0	0	0	0	0	0	0	0	0	0	0
Pol d 5	0	0	0.13	0	2.10	0	0	0	0	0	1.00	0
Pru p 1	0	0	0	0	0.57	0	0.16	0	2.91	0	0.17	0
Pru p 3	0	0	0	0	0	0	0	0	0	0	0	0
V Pru du 3	0	0	0	0	0	0	0	0	0	0	0	0
V Pru du 4	0	0	0	0	0	0	0	0	0	0	0	0
V Pru du 6	0	0	0	0	0	0	0	0	0	0	0	0
V Pru du 6.01	0	0	0	0	0	0	0	0	0	0	0	0
V Pru du 6.02	0	0	0	0	0	0	0	0	0	0	0	0
Sal k 1	0	0	0	0	0	0	0	0	0	0	0	0
Ses i 1	0	0	0	0	0	0	0	0	0	0	0	0
Tri a 14	0	0	0	0	0	0	0	0	0	0	0	0
Tri a 19.0101	0	0	0	0	0	0	0	0	0	0	0	0
Tri a aA_TI	0	0	0	0	0	0	0	0	0	0	0	0
V Tri a 36 191	0	0	0	0	0	0	0	0	3.69	0	0	0
V Tri a 36	0	0	0	0	0	0	0	0	0	0	0	0
V m43	0	0	0	0	0	0	0	0	0	0	0	0
V m82	0	0	0	0	0	0	0	0	0	0	0	0
Serine	0	0	0	0	0	0	0	0	0	0	0	0
Thioredoxin	0	0	0	0	0	0	0	0	0	0	0	0
Glutathione	0	0	0	0	0	0	0	0	0	0	0	0
peroxiredoxin	0	0	0	0	0	0	0	0	0	0	0	0
Profilin	0	0	0	0	0	0	0	0	0	0	0	0
Dehydrin	0	0	0	0	0	0	0	0	0	0	0	0
purothionin	0	0	0	0	0	0	0	0	0	0	0	0
LTP	0	0	0	0	0	0	0	0	0	0	0	0
V Ves v 1	0	0	0	0	0	0	0	0	0	0	0	0
Ves v 5	0	0	0	0	2.04	0	0	0	0	0	3.94	0
V Ves v 5	0	0	0	0	0	0	0	0	0	0	0	0

Patient	Donor 17		Donor 18		Donor 19		Donor 20		Donor 21		Donor 22		Donor 23	
Sample	PI	M	PI	M	PI	M	PI	M	PI	M	PI	M	PI	M
Method	IgE		IgE		IgE		IgE		IgE		IgE		IgE	
Act d 2	0	0	0	0	0	0	0	0	0	0	0	0	0	0
Act d 5	0.14	0	0	0	0	0	0	0	0	0	0	0	0	0
Act d 8	0	0	0	0	0	0	0	0	0	0	0	0	0	0
Aln g 1	0.36	0	0.21	0	3.34	0	0	0	0	0	0	0	4.05	0
Alt a 1	0	0	0	0	0	0	0	0	0	0	0	0	0	0
Alt a 6	0	0	0	0	0	0	0	0	0	0	0	0	0	0
Amb a 1	0	0	0	0	0	0	0	0	0	0	0	0	0	0
V Ana o 1	0	0	0	0	0	0	0	0	0	0	0	0	0	0
Ana o 2	0	0	0	0	0	0	0	0	0	0	0	0	0	0
V Ana o 2	0	0	0	0	0	0	0	0	0	0	0	0	0	0
V nAna o 2	0	0	0	0	0	0	0	0	0	0	0	0	0	0
V Ana o 3	0	0	0	0	0	0	0	0	0	0	0	0	0	0
Ani s 3	0	0	0	0	0	0	0	0	0	0	0	0	0	0
Api m 1	0	0	0	0	0	0	0	0	0	0	0	0	0.46	0
Api m 4	0	0	0	0	0	0	0	0	0	0	0	0	0	0
Ara h 1	0	0	0	0	0	0	0	0	0	0	0	0	0	0
Ara h 2	0	0	0	0	0	0	0	0	0	0	0	0	0	0
Ara h 3	0	0	0	0	0	0	0	0	0	0	0	0	0	0
Ara h 6	0	0	0	0	0	0	0	0	0	0	0	0	0	0
Ara h 8	0.77	0	0.28	0	0	0	0	0	0	0	0	0	2.90	0
Ara h 9	0	0	0	0	0	0	0	0	0	0	0	0	0	0
Art v 1	0	0	0.31	0	0.20	0	0.31	0	0	0	0	0	0	0
Art v 3	0	0	0	0	0	0	0	0	0	0	0	0	0	0
Asp f 1	0	0	0	0	0	0	0	0	0	0	0	0	0	0
Asp f 3	0	0	0	0	0	0	0	0	0	0	0	0	0	0
Asp f 6	0	0	0.32	0	0	0	0	0	0.13	0	0.18	0	0	0
Ber e 1	0	0	0	0	0	0	0	0	0	0	0	0	0	0
Bet v 1	5.55	0	3.02	0	15.08	0	0	0	0	0	0	0	24.92	0
Bet v 2	1.15	0	0	0	2.76	0	0	0	0	0	0	0	0	0
Bet v 4	0	0	0	0	0	0	0	0	0	0	0	0	0	0
Bla g 1	0	0	0	0	0	0	0	0	0	0	0	0	0	0
Bla g 2	0	0	0	0	0	0	0	0	0	0	0	0	0	0
Bla g 5	0	0	0	0	0.13	0	0	0	0	0	0	0	0	0
Bla g 7	0	0	0	0	0	0	0	0	0	0	0	0	0	0
Blo t 5	0	0	0	0	0	0	0	0	0	0	0	0	0	0
Bos d 4	0	0	0	0	0	0	0	0	0	0	0	0	0	0
V Bos d 4	0	0	0	0	0	0	0	0	0	0	0	0	0	0
Bos d 5	0	0	0	0	0	0	0	0	0	0	0	0	0	0
V Bos d 5	0	0	0	0	0	0	0	0	0	0	0	0	0	0
Bos d Lactoferrin	0	0	0	0	0	0	0	0	0	0	0	0	0	0
Bos d 8	0	0	0	0	0	0	0	0	0	0	0	0	0	0
V Bos d 8	0	0	0	0	0	0	0	0	0	0	0	0	0	0
aS1-casein	0	0	0	0	0	0	0	0	0	0	0	0	0	0
aS2-casein	0	0	0	0	0	0	0	0	0	0	0	0	0	0
b-casein	0	0	0	0	0	0	0	0	0	0	0	0	0	0
K-casein	0	0	0	0	0	0	0	0	0	0	0	0	0	0
Transferrin	0	0	0	0	0	0	0	0	0	0	0	0	0	0
Bos d 6	0	0	0	0	0	0	0	0	0	0	0	0	0	0
V BSA	0	0	0	0	0	0	0	0	0	0	0	0	0	0
Can f 1	0	0	0	0	15.17	0	0	0	0	0	0	0	0	0
Can f 2	0	0	0	0	0	0	0	0	0	0	0	0	0	0
Can f 3	0	0	0	0	0	0	0	0	0	0	0	0	0	0
V Can f 4	0	0	0	0	0	0	0	0	0	0	0	0	0	0
Can f 5	0	0	0	0	27.08	0.11	0	0	0	0	0	0	0	0
V Can f 5	0	0	0	0	0.19	0	0	0	0	0	0	0	0	0
V Can f 6	0	0	0	0	1.14	0	0	0	0	0	0	0	0	0
Che a 1	0	0	0	0	0	0	0	0	0	0	0	0	0	0
Cla h 8	0	0	0	0	0	0	0	0	0	0	0	0	0	0
Cor a 1.0401	1.25	0	0.92	0	4.74	0	0	0	0	0	0	0	5.16	0
Cor a 8	0	0	0	0	0	0	0	0	0	0	0	0	0	0
Cor a 9	0	0	0	0	0	0	0	0	0	0	0	0	0	0
Cry j 1	0	0	0	0	0	0	0	0	0	0	0	0	0	0
Cyn d 1	5.30	0	0	0	0	0	0	0	1.62	0	1.09	0	1.50	0
Cup a 1	0	0	0	0	0	0	0	0	0	0	0	0	0	0
Der f 1	0.15	0	7.18	0	0	0	9.88	0	0	0	0	0	3.84	0
Der f 2	0.33	0	0	0	0	0	27.46	0	0	0	0	0	14.89	0
Der p 1	0.16	0	0	0	0	0	6.76	0	0	0	0	0	2.20	0
Der p 2	0.49	0	0	0	0	0	21.64	0	0	0	0	0	13.02	0
V Der p 4	0	0	0	0	0	0	0	0	0	0	0	0	0	0
V Der p 5	0	0	0	0	0	0	0	0	0	0	0	0	0	0
V Der p 7	0	0	1.77	0	0	0	0	0	0	0	0	0	5.68	0
Der p 10	0	0	0	0	0	0	0	0	0	0	0	0	0	0
V Der p 11	0	0	0	0	0	0	0	0	0	0	0	0	0	0
V Der p 14	0	0	0	0	0	0	0	0	0	0	0	0	0	0
V Der p 15	0	0	0	0	0	0	0	0	0	0	0	0	0	0
V Der p 18	0	0	0	0	0	0	0	0	0	0	0	0	0	0
V Der p 21	0	0	1.15	0	0	0	0	0	0	0	0	0	0	0
V Der p 23	0.29	0	0	0	0	0	0	0	0	0	0	0	2.44	0
V clone 16	0	0	0.18	0	0	0	0	0	0	0	0	0	0	0

Patient	Donor 17		Donor 18		Donor 19		Donor 20		Donor 21		Donor 22		Donor 23	
Sample	PI	M	PI	M	PI	M	PI	M	PI	M	PI	M	PI	M
Method	IgE		IgE		IgE		IgE		IgE		IgE		IgE	
Equ c 1	0	0	0	0	4.41	0	0	0	0	0	0	0	0	0
Equ c 3	0	0	0	0	0	0	0	0	0	0	0	0	0	0
Fag e 2	0	0	0	0	0	0	0	0	0	0	0	0	0	0
Fel d 1	0.14	0	0	0	44.67	0.27	0	0	0	0	0	0	0	0
Fel d 2	0	0	0	0	0	0	0	0	0	0	0	0	0	0
Fel d 4	0	0	0	0	3.33	0	0	0	0	0	0	0	0	0
Gad c 1	0	0	0	0	0	0	0	0	0	0	0	0	0	0
Gal d 1	0	0	0	0	0	0	0	0	0	0	0	0	0	0
Gal d 2	0	0	0	0	0	0	0	0	0	0	0	0	0	0
Gal d 3	0	0	0	0	0	0	0	0	0	0	0	0	0	0
Gal d 5	0	0	0	0	0	0	0	0	0	0	0	0	0	0
Gly m 4	0.15	0	0	0	0	0	0	0	0	0	0	0	2.34	0
Gly m 5	0	0	0	0	0	0	0	0	0	0	0	0	0	0
Gly m 6	0	0	0	0	0	0	0	0	0	0	0	0	0	0
Hev b 1	0	0	0	0	0	0	0	0	0	0	0	0	0	0
Hev b 3	0	0	0	0	0	0	0	0	0	0	0	0	0	0
Hev b 5	0	0	0	0	0	0	0	0	0	0	0	0	0	0
Hev b 6.01	0	0	0	0	0	0	0	0	0	0	0	0	0	0
Hev b 8	1.31	0	0	0	0	0	0	0	0	0	0	0	0	0
Jug r 1	0	0	0	0	0	0	0	0	0	0	0	0	0	0
Jug r 2	0	0	0	0	0	0	0	0	0	0	0	0	0.57	0
Jug r 3	0	0	0	0	0	0	0	0	0	0	0	0	0	0
Lep d 2	0	0	0	0	0	0	0	0	0	0	0	0	0	0
Mal d 1	0.84	0	0.97	0	0.43	0	0	0	0	0	0	0	8.68	0
Mer a 1	2.10	0	0	0	0	0	0	0	0	0	0	0	0	0
Mus m 1	0	0	0	0	4.2	0	0	0	0	0	0	0	1.85	0
MUXF3	0	0	0	0	0	0	0	0	0	0	0	0	0	0
Ole e 1	0	0	0.49	0	0	0	0	0	0	0	0	0	0	0
Ole e 7	0	0	0	0	0	0	0	0	0	0	0	0	0	0
Ole e 9	0	0	0	0	0	0	0	0	0.20	0	0	0	0	0
Par j 2	0	0	0	0	0	0	0	0	0	0	0	0	0	0
Pen m 1	0	0	0	0	0	0	0	0	0	0	0	0	0	0
Pen m 2	0	0	0	0	0	0	0	0	0	0	0	0	0	0
Pen m 4	0	0	0	0	0	0	0	0	0	0	0	0	0	0
Phl p 1	19.64	0	0.30	0	2.10	0	0	0	2.82	0	1.38	0	0	0
Phl p 2	14.88	0	0	0	0	0	0	0	0	0	0	0	0	0
Phl p 4	5.86	0	0.16	0	0	0	0	0	2.66	0	1.60	0	2.06	0
Phl p 5	11.28	0	0	0	0	0	0	0	0	0	0	0	0	0
Phl p 6	12.30	0	0	0	0	0	0	0	0	0	0	0	0	0
Phl p 7	0	0	0	0	0	0	0	0	0	0	0	0	0	0
Phl p 11	0	0	0	0	0	0	0	0	0	0	0	0	0	0
Phl p 12	9.17	0	0	0	0	0	0	0	0	0	0	0	0	0
V Pis v 3	0	0	0	0	0	0	0	0	0	0	0	0	0	0
Pla a 1	0	0	0	0	0	0	0	0	0	0	0	0	0	0
Pla a 2	0	0	0	0	0	0	0	0	0	0	0	0	0.34	0
Pla a 3	0	0	0	0	0	0	0	0	0	0	0	0	0	0
Pla l 1	0	0	0	0	0	0	0	0	0	0	0	0	0	0
Pol d 5	0	0	0	0	0	0	0.14	0	0.14	0	0.24	0	4.96	0
Pru p 1	0.12	0	0.12	0	0	0	0	0	0	0	0	0	0.62	0
Pru p 3	0	0	0	0	0	0	0	0	0	0	0	0	0	0
V Pru du 3	0	0	0	0	0	0	0	0	0	0	0	0	0	0
V Pru du 4	0.79	0	0	0	0	0	0	0	0	0	0	0	0	0
V Pru du 6	0	0	0	0	0	0	0	0	0	0	0	0	0	0
V Pru du 6.01	0	0	0	0	0	0	0	0	0	0	0	0	0	0
V Pru du 6.02	0	0	0	0	0	0	0	0	0	0	0	0	0	0
Sal k 1	0	0	0	0	0	0	0	0	0.10	0	0	0	0	0
Ses i 1	0	0	0	0	0	0	0	0	0	0	0	0	0	0
Tri a 14	0	0	0	0	0	0	0	0	0	0	0	0	0	0
Tri a 19.0101	0	0	0	0	0	0	0	0	0	0	0	0	0	0
Tri a aA_TI	0	0	0	0	0	0	0	0	0	0	0	0	0	0
V Tri a 36 191	0	0	0	0	0	0	0	0	0	0	0	0	0	0
V Tri a 36	0	0	0	0	0	0	0	0	0	0	0	0	0	0
V m43	0	0	0	0	0	0	0	0	0	0	0	0	0	0
V m82	0	0	0	0	0	0	0	0	0	0	0	0	0	0
Serine	0	0	0	0	0	0	0	0	0	0	0	0	0	0
Thioredoxin	0	0	0	0	0	0	0	0	0	0	0	0	0	0
Glutathione	0	0	0	0	0	0	0	0	0	0	0	0	0	0
peroxiredoxin	0	0	0	0	0	0	0	0	0	0	0	0	0	0
Profilin	1.71	0	0	0	0	0	0	0	0	0	0	0	0	0
Dehydrin	0	0	0	0	0	0	0	0	0	0	0	0	0	0
purothionin	0	0	0	0	0	0	0	0	0	0	0	0	0	0
LTP	0	0	0	0	0	0	0	0	0	0	0	0	0	0
V Ves v 1	0	0	0	0	0	0	0	0	0	0	0	0	0	0
Ves v 5	0	0	0	0	0	0	0.30	0	0.15	0	0.25	0	2.88	0
V Ves v 5	0	0	0	0	0	0	0	0	0	0	0	0	0	0

Non-sensitized mothers (Donors 24-29)

Patient	Donor 24		Donor 25		Donor 26		Donor 27		Donor 28		Donor 29	
Sample	Pl	M	Pl	M	Pl	M	Pl	M	Pl	M	Pl	M
Method	IgE		IgE		IgE		IgE		IgE		IgE	
Act d 2		0	0	0	0	0	0	0	0	0	0	0
Act d 5	0	0	0	0	0	0	0	0	0	0	0	0
Act d 8	0	0	0	0	0	0	0	0	0	0	0	0
Aln g 1	0	0	0	0	0	0	0	0	0	0	0	0
Alt a 1	0	0	0	0	0	0	0	0	0	0	0	0
Alt a 6	0	0	0	0	0	0	0	0	0	0	0	0
Amb a 1	0	0	0	0	0	0	0	0	0	0	0	0
V Ana o 1	0	0	0	0	0	0	0	0	0	0	0	0
Ana o 2	0	0	0	0	0	0	0	0	0	0	0	0
V Ana o 2	0	0	0	0	0	0	0	0	0	0	0	0
V nAna o 2	0	0	0	0	0	0	0	0	0	0	0	0
V Ana o 3	0	0	0	0	0	0	0	0	0	0	0	0
Ani s 3	0	0	0	0	0	0	0	0	0	0	0	0
Api m 1	0	0	0	0	0	0	0	0	0	0	0	0
Api m 4	0	0	0	0	0	0	0	0	0	0	0	0
Ara h 1	0	0	0	0	0	0	0	0	0	0	0	0
Ara h 2	0	0	0	0	0	0	0	0	0	0	0	0
Ara h 3	0	0	0	0	0	0	0	0	0	0	0	0
Ara h 6	0	0	0	0	0	0	0	0	0	0	0	0
Ara h 8	0	0	0	0	0	0	0	0	0	0	0	0
Ara h 9	0	0	0	0	0	0	0	0	0	0	0	0
Art v 1	0	0	0	0	0	0	0	0	0	0	0	0
Art v 3	0	0	0	0	0	0	0	0	0	0	0	0
Asp f 1	0	0	0	0	0	0	0	0	0	0	0	0
Asp f 3	0	0	0	0	0	0	0	0	0	0	0	0
Asp f 6	0	0	0	0	0	0	0	0	0	0	0	0
Ber e 1	0	0	0	0	0	0	0	0	0	0	0	0
Bet v 1	0	0	0	0	0	0	0	0	0	0	0	0
Bet v 2	0	0	0	0	0	0	0	0	0	0	0	0
Bet v 4	0	0	0	0	0	0	0	0	0	0	0	0
Bla g 1	0	0	0	0	0	0	0	0	0	0	0	0
Bla g 2	0	0	0	0	0	0	0	0	0	0	0	0
Bla g 5	0	0	0	0	0	0	0	0	0	0	0	0
Bla g 7	0	0	0	0	0	0	0	0	0	0	0	0
Blo t 5	0	0	0	0	0	0	0	0	0	0	0	0
Bos d 4	0	0	0	0	0	0	0	0	0	0	0	0
V Bos d 4	0	0	0	0	0	0	0	0	0	0	0	0
Bos d 5	0	0	0	0	0	0	0	0	0	0	0	0
V Bos d 5	0	0	0	0	0	0	0	0	0	0	0	0
Bos d Lactoferrin	0	0	0	0	0	0	0	0	0	0	0	0
Bos d 8	0	0	0	0	0	0	0	0	0	0	0	0
V Bos d 8	0	0	0	0	0	0	0	0	0	0	0	0
aS1-casein	0	0	0	0	0	0	0	0	0	0	0	0
aS2-casein	0	0	0	0	0	0	0	0	0	0	0	0
b-casein	0	0	0	0	0	0	0	0	0	0	0	0
K-casein	0	0	0	0	0	0	0	0	0	0	0	0
Transferrin	0	0	0	0	0	0	0	0	0	0	0	0
Bos d 6	0	0	0	0	0	0	0	0	0	0	0	0
V BSA	0	0	0	0	0	0	0	0	0	0	0	0
Can f 1	0	0	0	0	0	0	0	0	0	0	0	0
Can f 2	0	0	0	0	0	0	0	0	0	0	0	0
Can f 3	0	0	0	0	0	0	0	0	0	0	0	0
V Can f 4	0	0	0	0	0	0	0	0	0	0	0	0
Can f 5	0	0	0	0	0	0	0	0	0	0	0	0
V Can f 5	0	0	0	0	0	0	0	0	0	0	0	0
V Can f 6	0	0	0	0	0	0	0	0	0	0	0	0
Che a 1	0	0	0	0	0	0	0	0	0	0	0	0
Cla h 8	0	0	0	0	0	0	0	0	0	0	0	0
Cor a 1.0401	0	0	0	0	0	0	0	0	0	0	0	0
Cor a 8	0	0	0	0	0	0	0	0	0	0	0	0
Cor a 9	0	0	0	0	0	0	0	0	0	0	0	0
Cry j 1	0	0	0	0	0	0	0	0	0	0	0	0
Cyn d 1	0	0	0	0	0	0	0	0	0	0	0	0
Cup a 1	0	0	0	0	0	0	0	0	0	0	0	0
Der f 1	0	0	0	0	0	0	0	0	0	0	0	0
Der f 2	0	0	0	0	0	0	0	0	0	0	0	0
Der p 1	0	0	0	0	0	0	0	0	0	0	0	0
Der p 2	0	0	0	0	0	0	0	0	0	0	0	0
V Der p 4	0	0	0	0	0	0	0	0	0	0	0	0
V Der p 5	0	0	0	0	0	0	0	0	0	0	0	0
V Der p 7	0	0	0	0	0	0	0	0	0	0	0	0
Der p 10	0	0	0	0	0	0	0	0	0	0	0	0
V Der p 11	0	0	0	0	0	0	0	0	0	0	0	0
V Der p 14	0	0	0	0	0	0	0	0	0	0	0	0
V Der p 15	0	0	0	0	0	0	0	0	0	0	0	0
V Der p 18	0	0	0	0	0	0	0	0	0	0	0	0
V Der p 21	0	0	0	0	0	0	0	0	0	0	0	0
V Der p 23	0	0	0	0	0	0	0	0	0	0	0	0
V clone 16	0	0	0	0	0	0	0	0	0	0	0	0

Patient	Donor 24		Donor 25		Donor 26		Donor 27		Donor 28		Donor 29	
Sample	Pl	M	Pl	M	Pl	M	Pl	M	Pl	M	Pl	M
Method	IgE		IgE		IgE		IgE		IgE		IgE	
Equ c 1	0	0	0	0	0	0	0	0	0	0	0	0
Equ c 3	0	0	0	0	0	0	0	0	0	0	0	0
Fag e 2	0	0	0	0	0	0	0	0	0	0	0	0
Fel d 1	0	0	0	0	0	0	0	0	0	0	0	0
Fel d 2	0	0	0	0	0	0	0	0	0	0	0	0
Fel d 4	0	0	0	0	0	0	0	0	0	0	0	0
Gad c 1	0	0	0	0	0	0	0	0	0	0	0	0
Gal d 1	0	0	0	0	0	0	0	0	0	0	0	0
Gal d 2	0	0	0	0	0	0	0	0	0	0	0	0
Gal d 3	0	0	0	0	0	0	0	0	0	0	0	0
Gal d 5	0	0	0	0	0	0	0	0	0	0	0	0
Gly m 4	0	0	0	0	0	0	0	0	0	0	0	0
Gly m 5	0	0	0	0	0	0	0	0	0	0	0	0
Gly m 6	0	0	0	0	0	0	0	0	0	0	0	0
Hev b 1	0	0	0	0	0	0	0	0	0	0	0	0
Hev b 3	0	0	0	0	0	0	0	0	0	0	0	0
Hev b 5	0	0	0	0	0	0	0	0	0	0	0	0
Hev b 6.01	0	0	0	0	0	0	0	0	0	0	0	0
Hev b 8	0	0	0	0	0	0	0	0	0	0	0	0
Jug r 1	0	0	0	0	0	0	0	0	0	0	0	0
Jug r 2	0	0	0	0	0	0	0	0	0	0	0	0
Jug r 3	0	0	0	0	0	0	0	0	0	0	0	0
Lep d 2	0	0	0	0	0	0	0	0	0	0	0	0
Mal d 1	0	0	0	0	0	0	0	0	0	0	0	0
Mer a 1	0	0	0	0	0	0	0	0	0	0	0	0
Mus m 1	0	0	0	0	0	0	0	0	0	0	0	0
MUXF3	0	0	0	0	0	0	0	0	0	0	0	0
Ole e 1	0	0	0	0	0	0	0	0	0	0	0	0
Ole e 7	0	0	0	0	0	0	0	0	0	0	0	0
Ole e 9	0	0	0	0	0	0	0	0	0	0	0	0
Par j 2	0	0	0	0	0	0	0	0	0	0	0	0
Pen m 1	0	0	0	0	0	0	0	0	0	0	0	0
Pen m 2	0	0	0	0	0	0	0	0	0	0	0	0
Pen m 4	0	0	0	0	0	0	0	0	0	0	0	0
Phl p 1	0	0	0	0	0	0	0	0	0	0	0	0
Phl p 2	0	0	0	0	0	0	0	0	0	0	0	0
Phl p 4	0	0	0	0	0	0	0	0	0	0	0	0
Phl p 5	0	0	0	0	0	0	0	0	0	0	0	0
Phl p 6	0	0	0	0	0	0	0	0	0	0	0	0
Phl p 7	0	0	0	0	0	0	0	0	0	0	0	0
Phl p 11	0	0	0	0	0	0	0	0	0	0	0	0
Phl p 12	0	0	0	0	0	0	0	0	0	0	0	0
V Pis v 3	0	0	0	0	0	0	0	0	0	0	0	0
Pla a 1	0	0	0	0	0	0	0	0	0	0	0	0
Pla a 2	0	0	0	0	0	0	0	0	0	0	0	0
Pla a 3	0	0	0	0	0	0	0	0	0	0	0	0
Pla l 1	0	0	0	0	0	0	0	0	0	0	0	0
Pol d 5	0	0	0	0	0	0	0	0	0	0	0	0
Pru p 1	0	0	0	0	0	0	0	0	0	0	0	0
Pru p 3	0	0	0	0	0	0	0	0	0	0	0	0
V Pru du 3	0	0	0	0	0	0	0	0	0	0	0	0
V Pru du 4	0	0	0	0	0	0	0	0	0	0	0	0
V Pru du 6	0	0	0	0	0	0	0	0	0	0	0	0
V Pru du 6.01	0	0	0	0	0	0	0	0	0	0	0	0
V Pru du 6.02	0	0	0	0	0	0	0	0	0	0	0	0
Sal k 1	0	0	0	0	0	0	0	0	0	0	0	0
Ses i 1	0	0	0	0	0	0	0	0	0	0	0	0
Tri a 14	0	0	0	0	0	0	0	0	0	0	0	0
Tri a 19.0101	0	0	0	0	0	0	0	0	0	0	0	0
Tri a aA_TI	0	0	0	0	0	0	0	0	0	0	0	0
V Tri a 36 191	0	0	0	0	0	0	0	0	0	0	0	0
V Tri a 36	0	0	0	0	0	0	0	0	0	0	0	0
V m43	0	0	0	0	0	0	0	0	0	0	0	0
V m82	0	0	0	0	0	0	0	0	0	0	0	0
Serine	0	0	0	0	0	0	0	0	0	0	0	0
Thioredoxin	0	0	0	0	0	0	0	0	0	0	0	0
Glutathione	0	0	0	0	0	0	0	0	0	0	0	0
peroxiredoxin	0	0	0	0	0	0	0	0	0	0	0	0
Profilin	0	0	0	0	0	0	0	0	0	0	0	0
Dehydrin	0	0	0	0	0	0	0	0	0	0	0	0
purothionin	0	0	0	0	0	0	0	0	0	0	0	0
LTP	0	0	0	0	0	0	0	0	0	0	0	0
V Ves v 1	0	0	0	0	0	0	0	0	0	0	0	0
Ves v 5	0	0	0	0	0	0	0	0	0	0	0	0
V Ves v 5	0	0	0	0	0	0	0	0	0	0	0	0
